# Engaging Communities to Address the Opioid Crisis in Ohio: Lessons Learned

**DOI:** 10.18061/ojph.6411

**Published:** 2026-03-24

**Authors:** Amy Farmer, Brittany Joseph, Kendra Green, Owusua Yamoah, Kathleen Egan, Brandon Elmore, James Fye, Tara Crawford, Laura Ashley Wilson, Judy Harness, Sara M. Roberts, Bridget Freisthler, Pamela Salsberry

**Affiliations:** 1 College of Medicine, The Ohio State University, Columbus, OH; 2 Mary Ann Swetland Center for Environmental Health, Case Western Reserve School of Medicine, Cleveland, OH; 3 Division of Public Health Services, Wake Forest University School of Medicine, Winston-Salem, NC; 4 ERIK | Translational Data Analytics Institute, The Ohio State University, Columbus, OH; 5 Ohio Colleges of Medicine Government Resource Center, The Ohio State University, Columbus, OH; 6 Case Western Reserve University School of Medicine, Cleveland, OH; 7 College of Social Work, University of Tennessee, Knoxville, TN; 8 College of Public Health, The Ohio State University, Columbus, OH

**Keywords:** Community engagement, Opioid use disorder (OUD), Stigma reduction, People with lived experience (PWLE), Coalition building

## Abstract

Community engagement is considered essential in addressing community health problems. This paper details lessons learned from community engagement efforts implemented to reduce opioid overdose deaths in 9 Ohio communities participating in wave 2 of the HEALing (Helping to End Addiction Long-term) Communities Study (HCS). We describe community engagement strategies implemented during the wave 2 intervention period, including coalition-building, fostering community collaboration, prioritizing health equity and policy advocacy, stigma reduction education, and elevating voices of people with lived experience (PWLE). We provide reflections from the perspective of community engagement facilitators (CEFs) on how these strategies informed the implementation of evidence-based practices (EBPs) within communities. Our community engagement approaches and reflections highlight 3 key lessons for future community engagement efforts: 1) an intentional and constant focus on community collaboration is a necessary and essential ingredient for successful community engagement; 2) introducing opportunities to define and discuss equity within the local community can lead to recognition of subgroups for directed intervention; and 3) engaging PWLE can foster trust around stigmatizing issues.

## INTRODUCTION

In 2018, opioid deaths accounted for more than one-third of all drug overdose-related deaths in the United States.^1^ Along with increased prevalence of opioid misuse, the rapidly changing landscape of the opioid crisis from prescription pills to heroin and now fentanyl has increased the need for community level supports to intervene in this epidemic.^2^ To address this crisis, the HEALing (Helping to End Addiction Long-term) Communities Study (HCS) was implemented in 67 communities across 4 states (Kentucky, Massachusetts, New York, and Ohio). The HCS was a parallel-arm, wait-list controlled, cluster-randomized trial aimed to reduce opioid overdose deaths in highly affected communities through the Communities That HEAL (CTH) intervention.^3^ This study protocol (Pro00038088) was approved by Advarra Inc, the HEALing Communities Study single institutional review board.

The CTH intervention was grounded in community coalitions that worked through a multiphase process to select evidence-based practices (EBPs) and stigma-reducing communication campaigns.^4–6^ A critical component of the CTH intervention was to employ field staff with expertise in community engagement, data use for decision making, and implementation of the opioid overdose reduction continuum of care approach (ORCCA).^7,8^ In Ohio, each HCS field team consisted of 3 individuals, including a community engagement facilitator (CEF), a data coordinator, and an intervention facilitator. This team worked in the local community alongside local coalitions to implement the CTH intervention.

Throughout the study, field teams were directed to adapt the CTH intervention protocol to the local community’s norms, traditions, and culture.^4^ Field staff used various strategies to tailor the intervention to align with local needs, thus working to achieve the goals of the coalitions and meet the study milestones.

This paper builds on current HCS literature examining the CTH intervention by analyzing the CEF’s community engagement efforts within the 9 Ohio wait-listed control HCS communities, referred to as the wave 2 communities, during the intervention phase of July 2022 through December 2023.^4,9^ From the outset of the research, the role of the CEF was to lead the community engagement efforts of the field team by focusing on relationships within the coalition and with the broader community. This engagement was critical throughout CTH implementation as it provided critical context and structure to the flow of the research protocol. The aim of this research was to reflect, using the Reflective Analysis Tool (Table 1), on the engagement efforts completed throughout the intervention period and, through this reflection, identify lessons learned about the processes, successes, and challenges of doing this work.

### Background: Brief Overview of the Communities That HEAL Intervention in Ohio

The CTH intervention included 7 phases designed to guide communities through a systematic process with the goal of selecting and implementing evidence-based strategies with the potential for reducing opioid overdose deaths in their communities. The phases were 0) preparation, 1) getting started, 2) getting organized, 3) community profiles and data dashboards, 4) community action planning, 5) implementation and monitoring, and 6) sustainability planning.^4^ During the implementation of the intervention in wave 2, there was an intentional national focus on addressing local equity issues in the treatment and recovery of those with substance use disorder. These equity discussions occurred throughout all phases and centered on identifying groups with limited access to care in their communities. Specifically, the following special populations were identified as groups with limited access to care: persons of color, those who were homeless, unemployed, uninsured, persons with a disability, LGBTQ+, pregnant women, and those with low income.

#### Description and Responsibilities of Community Engagement Facilitators

The role of the CEF was to lead the engagement efforts of the field team by forming relationships early with both the coalition and the broader community. The CEFs were identified and joined the field team primarily during phase 0 (preparation) of the intervention. The goal was to hire individuals already integrated within the community, or with knowledge of similar counties, to support the project in all phases of the intervention. A key structural component of this role was its positionality, embedding a research team member within the communities, allowing direct communication between coalitions and researchers.

The CEFs were expected to demonstrate skills in relationship development, community engagement processes, and conflict resolution. They served as the “boots on the ground” for the broader research team. Each CEF was responsible for representing the HCS project to the community, facilitating organizational and community discussions around the project and interventions, and assisting research staff to engage community stakeholders and members in discussions related to the project and its implementation.

The CEFs supported needs assessment, facilitated consensus-building among community stakeholders on strategy selection to reduce opioid overdose deaths, fostered trust in collaborative relationships, offered contextual insight to the research team, assisted with fidelity monitoring, ensured sustained focus on health equity, and worked with communities to develop sustainability plans.

## METHODS

### Sample

The 9 wave 2 coalitions had focused on various health issues before HCS entered the counties. Three of the coalitions were focused on substance use treatment and recovery, one on opioid treatment and overdose reduction, two on substance use treatment and recovery with primary prevention, two on primary substance use prevention, and one on general population health. Once the HCS intervention began, discussions occurred between coalition leadership and HCS county field staff to determine a coalition structure that would support the HCS intervention. In 6 of the counties, the decision was made to create a subcommittee of the original coalition, with decision making authority residing with the subcommittee. One of the counties chose to use the preexisting coalition, without changes to the structure. One county formed a completely new coalition, and another county chose a subcommittee structure but required reporting back to the preexisting coalition. The CEFs leveraged their expertise in data driven science and community engagement to influence coalition leadership toward inclusion of individuals most affected by the crisis, as well as other populations and sectors necessary, to implement the CTH intervention and achieve HCS goals, in the coalition.

### Materials

Table 2 displays the tools used to gather and track data related to engagement with coalitions and communities.

### Analysis

Reflective analysis, based on questions detailed in Table 1, was used to examine the efforts of CEFs throughout wave 2. These questions were answered individually by each CEF in an online shared document, then discussed further in a series of virtual meetings which included CEFs, program managers, and faculty leaders, ultimately narrowing the focus to several key concepts for further development, discussion, and manuscript development.

Two questions emerged as the impetus for development of this manuscript: “What lessons did we learn from our community engagement efforts throughout this study?” and “What insights can be shared with others engaged in this type of work?” The CEFs continued to work with program managers and faculty leaders throughout the development process, refining and editing their work in shared virtual spaces to accurately represent the experiences and lessons deemed most important to share with the broader research community.

## RESULTS

Two broad themes, encompassing the topics noted in Table 1, emerged from the review and analysis of the CEF’s reflections of their activities. One theme centered on the significant efforts to foster community collaboration to reduce opioid overdose deaths and the second theme was associated with developing an equity-based approach to responding to the opioid crisis. Below are the lessons learned by the CEFs in implementing the CTH model, highlighting the processes used, and the successes and challenges encountered. These findings provide information for future community engagement efforts seeking to address public health issues such as the opioid crisis.

### Fostering Community Collaboration

Coalition building, a well-documented community engagement strategy for planning, executing, adapting, and implementing health prevention and promotion research, was utilized across HCS communities in all phases of the CTH intervention.^12,13^ The community coalition action theory (CCAT), developed in 2002 by Butterfoss and Kegler, describes how community coalitions impact health and social outcomes such as opioid use disorder (OUD) and move through a “life cycle” of formation, maintenance, and sustainment.^14^ According to CCAT, the constructs of coalition membership, leadership and staffing, structures, operations and processes, pooled resources, member engagement, assessment and planning, and community context enhance coalition function and lead to the implementation of strategies that have the potential for community change outcomes; for example, increasing community capacity, and impacting health and social outcomes.^14^ Using the CCAT as a framework, we discuss 3 constructs as identified by Butterfoss and Kegler: coalition membership, coalition structures, and processes of operations.^14^

#### Coalition Membership

To enhance coalition membership, CEFs utilized tools to understand community context, including windshield surveys and listening tours, to immerse themselves in the community and identify stakeholders not involved in or engaged with the coalition. Throughout the listening tours, CEFs reached out to entities who interacted with individuals in recovery including treatment providers, support groups, mental health treatment organizations, law enforcement, judicial systems, first responders, jails/prisons, job and family services offices, recovery housing providers, faith-based organizations, and employers. This outreach was partially facilitated by warm introductory email messages from existing coalition leadership in each respective county, but much of it involved making “cold calls.” These initial contacts led to virtual or in-person meetings to learn more about each organization and encourage participation in the coalition. The goal of the listening tours was to understand the opioid crisis from the perspective of local community members, coalition members, and organizations and to ask the question, “What does the opioid crisis look like in your community?” Some of these interactions led to networking opportunities with other individuals and organizations central to the conversation in the community.

The HCS staff identified a gap in involvement of people with lived experience (PWLE) in coalition membership; only 4 of the 9 coalitions included PWLE as part of their membership before HCS involvement. The CEFs encouraged coalitions to include PWLE in decision-making and discussions, including those who currently or formerly used opioids, their family members, and others who could speak to the needs, challenges, and preferences related to their first-hand experience. As a result of CEF efforts, three additional coalitions added PWLE to their membership during the study. Bringing PWLE into community coalition meetings where their views and ideas were valued empowered them to contribute toward collective goals from their unique perspectives and experiences. Their involvement challenged coalition members to think outside the box, leading to innovative solutions that may not have been considered otherwise. For example, PWLE provided insight into barriers to accessing harm reduction services, offered practical recommendations to improve outreach efforts, and helped shape messaging in ways that reduced stigma and resonated with those directly impacted. Their participation ensured that coalition efforts were not only well-intentioned but also practical, relevant, and responsive to the real needs of the community.

One way that PWLE were successfully engaged in the CTH intervention was through Photovoice projects to highlight local issues impacting those with lived experience by sharing personal photographs and stories. Three HCS wave 2 coalitions voluntarily participated in Photovoice projects involving PWLE.^15–17^ The CEFs assisted with recruitment and engagement of participants through local networks; subsequently, the results of those projects, including themes highlighting local barriers and facilitators to the recovery process through the lens of local PWLE, were shared with local coalitions to better inform EBP selection and refinement.^15^

#### Coalition Structure, Operations, and Processes

As CEFs began working within the counties, it became clear that many organizations were contributing valuable efforts toward the reduction of opioid overdose deaths, but in silos. This approach, at times influenced by competition among agencies offering similar services to patients with OUD, had the potential to negatively impact the work of HCS in the community. One approach to addressing this was to bring partners together toward a more collaborative and evidence-based approach made possible by ensuring that invitations to join the coalition were extended to everyone who could contribute to their work, including PWLE.

A critical factor in ensuring power sharing and equitable partnerships in HCS involved examining the mission and/or value statements of each coalition and incorporating those elements into a formal partnership agreement between each coalition and HCS. The CEFs supported coalitions in establishing standard procedures for their operation including a formal process to record and publish meeting minutes, distribute meeting agendas, and vote on proposed actions. These processes were subsequently discussed and implemented into the partnership agreement.^4^

Most coalitions chose to identify key individuals, designated as “champions,” to work more closely with HCS staff throughout the study. Key champion roles included coalition lead, communications, EBP, data, and PWLE. A successful strategy utilized in coalition building and outlined by Gilgoff et al included compensating coalition champions and leaders financially with a small monthly stipend provided through the study.^18^ The expectations for engagement and involvement by champions were greater than for other coalition members. In some counties, additional workgroups were also established to focus on specific coalition interests. These specific workgroups included media and communications, ORCCA/EBP intervention, and data.

Decision-making power and responsibilities, including financial decisions about study funding and the selection of EBPs for implementation, were managed differently by each coalition, and CEFs facilitated discussions to encourage consensus building and assist coalitions in structuring their processes. Each county received funds to implement EBPs related to opioid education and naloxone distribution, medication treatment for opioid use disorder, and safer opioid prescribing and dispensing. The actual decision-making and approval process varied widely with some coalitions voting by consensus, others by simple majority, and still others by allocating voting power to smaller workgroups.

One barrier to coalition building was the stigmatization of people with OUD in the community. Stigma and related bias, such as the “not in my backyard” mentality, can lead to social isolation of individuals with substance use disorder and even prevent them from seeking help.^19^ An example of stigma that occurred in some HCS counties was agencies’ unwillingness to participate in the coalition because of controversial opinions held by their leadership about individuals with OUD.

Unraveling barriers to collaboration took time and effort but encouraged transparency and promoted honesty among coalition members. In one county, a coalition member stepped forward during a conversation about medications for opioid use disorder (MOUD) and shared that they had been in recovery for many years. Sharing their personal experience allowed other coalition members to view OUD treatment from a different perspective.

### Applying the Lens of Health Equity

Community engagement has been identified as a best practice for developing approaches to address health inequities.^20^ The Ohio team’s plan for health equity “acknowledge[d] that structural barriers influence outcomes for marginalized populations…, that each Ohio HCS community had its own health equity priorities, and [that] each community started from a different place regarding understanding inequities.”^21^ This understanding meant that strategies to achieve equitable health could vary across HCS counties and included examination of data on overdoses by subgroup where available (sex, homeless status, race, ethnicity, socioeconomic status, insurance status) and understanding the reach of treatment programs.

#### Prioritizing Health Equity

Within wave 2 of HCS, the initial approach to ensuring health equity while engaging with communities was to identify inequities in access to recovery and treatment services for underserved and/or underrepresented populations. These populations included but were not limited to persons of color, those who were homeless, unemployed, uninsured, persons with a disability, LGBTQ+, pregnant women, and those with low income. Following this initial assessment, CEFs prioritized health equity in their engagement with the goal of making interventions more culturally sensitive, locally relevant, accessible, and sustainable.

One way that CEFs prioritized health equity was to encourage the coalition and community partners to embrace PWLE guidance in decision-making to help understand and address the inequities in access to treatment and recovery services. This proved to be a daunting task at times due to difficulty in identifying and accessing PWLE. The CEFs engaged PWLE by relying on local organizations with existing relationships. For instance, the CEF of a county attended recovery meetings and, through relationship-building at these meetings, was able to invite PWLE to coalition meetings to encourage dialogue about OUD in the community.

Another strategy toward health equity was expanding HCS reach to include non-English speakers. All HCS Ohio communication materials were originally only available in English; however, some counties had Spanish-speaking and Somali-speaking populations. Materials were adapted to reach these populations. Developing accessible communication campaign materials allowed CEFs to advance health equity by engaging with marginalized groups in the community.

Through initial assessments in some counties, CEFs identified transportation as a major barrier for achieving health equity because it limited access to treatment for some individuals. To address this barrier, several counties purchased mobile health units to bring resources to low-income, high-risk populations across entire counties. One county also worked with a local treatment agency, the only methadone clinic in the county, to purchase a large passenger vehicle to transport clients to and from their appointments. Other strategies included providing vouchers for gas cards to assist low-income populations with the cost of travel. These approaches are in stark contrast to how services and resources, including MOUD, have traditionally been received by clients who are expected, despite transportation barriers, to travel to provider offices for treatment.

#### Policy Advocacy

Policy advocacy within the context of this work helped to identify systemic issues within the policies, practices, laws, and bylaws of businesses, organizations, and government entities that create barriers to recovery and, often unintentionally, perpetuate stigma toward those with OUD. The CEFs made concentrated efforts to identify potentially problematic policies by using active listening during meetings, engaging in one-on-one conversations with community stakeholders, and guiding discussions to uncover issues. Throughout HCS, policies identified by the coalitions were tracked in a database and routed to local, state, and federal agencies with the capacity to facilitate change where needed. By the end of the wave 2 period, 48 policies that create barriers to recovery or perpetuate stigma had been identified across the 9 counties and routed to those in positions of power to amend them.

One example involved challenges with local and state policies that did not allow the installation of NaloxBoxes at a highway rest area, despite data showing the area was a hot spot for overdose activity. Through engagement and collaboration with policy makers and state partners, 2 NaloxBoxes were successfully installed within that county, which subsequently led to efforts by Ohio’s governor’s office to install NaloxBoxes in 65 interstate highway rest areas across the state of Ohio.^22^ Because these policy barriers were recognized and addressed, due in large part to the success of engaging across sectors, significant barriers were removed and a key strategy was implemented.

The unique perspectives of PWLE on local issues and policies influencing employment, housing, law enforcement interactions, and judicial practices provided opportunities for discussions about areas where change could be accomplished at the community level. Additionally, CEFs asked coalition-involved organizations to examine their own policies and procedures to identify areas where common business practices might create barriers for successful implementation of EBPs.

### Discussion and Implications

The approaches adapted by the CEFs challenged the common hierarchical structure of experts working outside of communities.^23^ These new approaches facilitated bringing together community members and key stakeholders to create sustainable and equitable relationships that enhanced community ownership of the HCS coalitions and the selection and implementation of EBP strategies to address OUD and overdose deaths. Within coalitions, diversity in membership has been recognized as an asset for leveraging public support when addressing health concerns.^24^ Kania and Kramer assert that interventions that only engage individual organizations cannot result in large-scale social change.^25^ By fostering collaboration among diverse stakeholders, HCS prioritized cross sector partnerships. The kind of community engagement needed to foster and sustain these relationships required time and resources; specifically full-time staff dedicated to community engagement. Additionally, commitment from all parties involved was necessary for developing trusting relationships over time. We found that this community-engaged approach promoted community ownership and recognition of OUD as a problem that impacted the community rather than only impacting “them” as a group of outsiders with a problem. Ahmed and Palermo argue that when community engagement is done the right way, it can “help mobilize resources and influence systems, change relationships among partners, and serve as catalysts for changing policies, programs, and practices.”^26^ This “agent of change” mentality supports the idea that coalitions exist, at least in part, to drive change at the local level by leveraging the relationships between partner organizations and drawing upon their collective influence to positively affect desired outcomes.

A focus on general health equity was needed throughout the study period. A major effort of the engagement staff was to encourage a broadening of the coalition membership to include people most affected by the opioid epidemic. As the voices of PWLE have historically been excluded from research and policies that directly impact them, significant outreach to include these voices occurred.^27^ This was critical as Thornicroft et al argue PWLE are key change agents in reducing stigma related to mental health and, as such, must be at the forefront of identifying and implementing solutions.^28^ During the study, CEFs encouraged PWLE engagement in goal setting and the development of solutions to help reduce stigma, both toward those with OUD and toward evidence-based treatment options. Through Photovoice, PWLE engaged with policy makers and informed solutions that impacted them directly.^15^ The PWLE not only brought their experiences with OUD but also local knowledge valued by coalition members and in CBPR.^15,29^ We further observed that PWLE found the opportunity to be included in decision-making empowering. It was clear to those in the CEF role that individuals with lived experience have a role to play in coalitions either as active participants or as consultants to those with the power to affect positive change on their behalf. This aligns with observations by other researchers who found that when they engaged PWLE in their research, the outcome better reflected the needs of the population for which the research was designed, resulting in more successful adoption, implementation, and sustainability.^27,28,30^

Despite differences in demographics, population density, and coalition makeup (eg, coalitions comprised of drug prevention and treatment organizations only versus those with more diverse membership including health care, criminal justice, faith-based organizations, first responders, local businesses, etc.), CEFs discovered that similar approaches to working with coalitions yielded comparable results in each of the key areas noted above. These areas of focus represent lessons learned by CEFs throughout the HCS implementation and facilitated the selection of the most promising EBPs for their communities.

### Strengths and Limitations

#### Strengths

In Ohio, HCS implemented the same protocol across all 9 counties. These counties were of varied sizes and geographies but received similar resources in staffing and funding during the intervention period. This study design adds to the generalizability of the results. While CEFs came to this study with different experiences regarding community engagement and knowledge of OUD, these differences were addressed through rigorous training in both substance use disorder and community engagement. Additionally, tailored support was provided through weekly meetings with field team managers and faculty leads, and additional training continued throughout the tenure of each CEF.

#### Limitations

As noted above, these findings were based on 9 communities in Ohio. A larger sample, inclusive of other coalition structures that may have different processes, could face different challenges. Furthermore, involving communities during study conceptualization and design may have resulted in fewer barriers. Finally, changes in staffing and/or leadership at partner organizations sometimes created challenges for engagement and gaps of expertise within the coalition. Conversely, when new members joined, HCS staff spent time orienting, building relationships, and establishing trust with new coalition members while the intervention was in progress.

## PUBLIC HEALTH IMPLICATIONS

Our community engagement approaches and reflections highlight 3 key lessons for future community engagement efforts: 1) an intentional and constant focus on community collaboration is a necessary and essential ingredient for successful community engagement; 2) introducing opportunities to define and discuss equity within the local community can lead to recognition of subgroups for directed intervention; 3) engaging PWLE can foster trust around stigmatizing issues.

These findings have practical implications for coalitions and public health professionals seeking a more structured approach to coalition-level work and reflect the consensus of community engagement professionals with “boots on the ground” experience. Understanding the strategies that were effective in these 9 Ohio communities may contribute to the development of scalable and replicable community engagement models for addressing broader public health issues.

## Figures and Tables

**Table 1.** Reflective Analysis Tool for Community Engagement Facilitators

Reflection Topic	Specific Considerations
Community engagement	How did community engagement evolve in your county? What tools did you use to facilitate engagement and collaboration?
Communications	How did you achieve communications campaigns in your county? What media were most successful? What challenges did you encounter?
Health equity	What areas of health inequity were most prevalent in your county? Where were improvements needed? How did you address health inequity throughout HCS?
Policy research	What was your approach to discovering policy issues that might impact the HCS efforts in your county? What policy issues were identified?
Stigma reduction	How was stigma addressed within the coalition and community you worked in? Was it only addressed within the context of communications campaigns or were there presentations or learning opportunities focused on stigma reduction?
People with lived experience (PWLE)	In what ways were PWLE included in the coalition prior to HEALing Communities Study (HCS) involvement? Did this evolve or change during your time in the county?
Community-based participatory research (CBPR)	How were the concepts of CBPR implemented into your approach to community engagement in your county?

**Table 2.** Data Collection Tools

Data Collection Tool	Purpose
Windshield surveys^10^	Allowed community-based research staff to see and understand the physical layout of the community, including factors influencing health equity and social determinants of health.
Listening tours^11^	Initial meetings with coalition members and other members of the community to gain insight and understanding of the opioid crisis from the local perspective.
Case notes	Completed by the field staff in a shared database and used to collect information about interactions between community and coalition members and community-based research staff, including their observations of interactions in the community.
Coalition meeting minutes	Used to keep an accurate record of the HEALing Communities Study (HCS) coalition meetings.
Weekly staff meetings	Weekly meetings with field team members, program managers, and faculty leads to discuss, plan, and problem-solve Communities That HEAL (CTH) implementation in local communities.
Reflective data analysis	Post-intervention, used as a tool for community engagement facilitator (CEF) self-analysis of the impact of community engagement efforts, including the emergence of common themes and lessons learned.
